# Rapid mapping of seasonal malaria transmission risk for strategic elimination planning in Swaziland

**DOI:** 10.1186/1475-2875-11-S1-O7

**Published:** 2012-10-15

**Authors:** Justin M Cohen, Sabelo Dlamini, Joe Novotny, Deepika Kandula, Simon Kunene, Andrew J Tatem

**Affiliations:** 1 Clinton Health Access Initiative, Boston, MA, USA; 2 National Malaria Control Programme, Mbabane, Swaziland; 3 University of Florida, Gainesville, FL, USA

## Background

As successful malaria control programs move towards elimination, they must identify residual transmission foci, focus on both asymptomatic and symptomatic infections, and manage importation risk. High spatial and temporal resolution maps of malaria risk can support all of these activities, but new approaches are required to provide accurate case-based risk maps for very low prevalence countries like Swaziland, where fewer than 500 cases were reported in 2011.

## Materials and methods

Household locations and travel histories of confirmed malaria patients were recorded through routine surveillance by the Swaziland National Malaria Control Programme for the higher transmission months of Jan to Apr 2011 and the lower transmission months of May to Dec. Household locations with locally-acquired infections were compared against a random set of background points with respect to variables related to environment, population density, vector control, and distance to the households of imported cases. Comparisons were made separately for the high and low transmission seasons. The regression tree classification approach Random Forest was used to generate maps predicting the probability of a locally-acquired case at 100 m resolution across Swaziland during the high and low transmission seasons.

## Results

Results indicated that case households during the high transmission season tended to be located at lower elevations, closer to stream channels, in more sparsely populated areas, with higher rainfall and lower temperature than random background points (all p < 0.01). No significant difference was evident with distance to the nearest imported case household. Similar differences were evident during the low transmission season, but environmental variables like distance to stream channels and water bodies were no longer significantly different, while low season case households were located significantly nearer to those of imported cases (p = 0.02). Maps from the fit models suggested better predictive ability during the high season.

## Conclusions

The rapid, high-resolution mapping approaches described here appear useful for helping elimination programs understand the epidemiology of a disappearing disease. Generating case-based risk maps at high spatial and temporal resolution will allow control programs to direct interventions in response to evidence-based measures of risk and ensure that the impact of limited resources is maximized to achieve and maintain malaria elimination.

